# Circulating levels of microRNA193a-5p predict outcome in early stage hepatocellular carcinoma

**DOI:** 10.1371/journal.pone.0239386

**Published:** 2020-09-22

**Authors:** Sven H. Loosen, Theresa H. Wirtz, Sanchari Roy, Mihael Vucur, Mirco Castoldi, Anne T. Schneider, Christiane Koppe, Tom F. Ulmer, Anjali A. Roeth, Jan Bednarsch, Patrick H. Alizai, Pia Paffenholz, Münevver Demir, Christian Trautwein, Frank Tacke, Ulf P. Neumann, Christoph Roderburg, Tom Luedde

**Affiliations:** 1 Clinic for Gastroenterology, Hepatology and Infectious Diseases, University Hospital Düsseldorf, Medical Faculty of Heinrich Heine University Düsseldorf, Düsseldorf, Germany; 2 Department of Medicine III, University Hospital RWTH Aachen, Aachen, Germany; 3 Division of Gastroenterology, Hepatology and Hepatobiliary Oncology, University Hospital RWTH Aachen, Aachen, Germany; 4 Department of Visceral and Transplantation Surgery, University Hospital RWTH Aachen, Aachen, Germany; 5 Department of Urology, University Hospital Cologne, Cologne, Germany; 6 Department of Hepatology and Gastroenterology, Charité University Medicine Berlin, Berlin, Germany; University of Navarra School of Medicine and Center for Applied Medical Research (CIMA), SPAIN

## Abstract

While tumor resection and liver transplantation (LT) represent potentially curative therapeutic options for patients with early-stage hepatocellular carcinoma (HCC), the identification of the ideal surgical candidates has remained challenging. Just recently, miRNA-193a-5p was described as a tumor suppressor in murine and human HCC but only little is known about circulating miRNA-193a-5p in HCC patients. Here, we evaluated serum levels of miR-193a-5p by qPCR in 41 HCC patients undergoing tumor resection (n = 33) or LT (n = 8) and 20 controls. Circulating relative miR-193a-5p levels were significantly elevated in HCC patients compared to healthy controls. While relative miR-193a-5p levels were comparable between patients of different underlying disease etiology and tumor size, high relative miR-193a-5p levels were predictive for the patients’ postoperative outcome, which was confirmed in uni- and multivariate Cox-regression analysis. As such, HCC patients with a preoperative relative miR-193a-5p level above the ideal cut-off value (3.57) had a median overall survival (OS) of only 451 days compared to 1158 days in patients with a relative miR-193a-5p level below this cut-off value. Our data support a novel function of miR-193a-5p as a biomarker in early-stage HCC patients that might help to identify the best surgical candidates in terms of postoperative outcome.

## Introduction

Hepatocellular carcinoma (HCC) represents the most common primary liver cancer and is associated with rising incidence rates [1]. While the majority of patients initially present with an advanced non-resectable tumor stage (BCLC B or C) and are assigned to systemic chemotherapy or local ablative approaches (e.g. transarterial chemoembolization (TACE)), curatively indented surgical tumor resection as well as liver transplantation (LT) represent the standard of care for early stage HCC patients (BCLC 0 or A) [2]. However, despite receiving a complete tumor resection around 70% of patients develop disease recurrence within five years [3]. Moreover, surgical resection is related to postoperative complications including liver failure, infections and delirium, which might be associated with a prolonged hospital stay as well as an unfavourable outcome after surgery [4]. Despite several prognostic algorithms and preoperative assessment strategies (including laboratory parameters, imaging techniques as well as the patients’ clinical performance status) have been proposed so far [5], identification of patients that particularly benefit from liver resection or LT in terms of overall survival (OS) still represent a major clinical challenge.

Micro RNAs (miRNA) are a group of small RNA that regulate gene expression on a posttranscriptional and posttranslational level [6]. During the past decade, miRNAs were established as both tumor suppressors and oncogenes in different malignancies including HCC [7, 8]. We recently identified down-regulation of miR-193a-5p as a common feature of murine and human HCC regardless of the disease etiology [9]. By reducing levels of nucleolar and spindle-associated protein 1 (NUSAP-1), miR-193a-5p prevented the development of HCC and lower expression of miR-193a-5p was directly associated with shorter survival times of patients. Besides their function as intracellular regulators of gene expression, miRNAs are increasingly used as blood-based biomarkers for manifold biological processes. In HCC, various circulating miRNAs were proposed as both diagnostic and prognostic markers [10, 11]. However, the use of these miRNAs in clinical routine was hampered by a tremendous lack in data standardization, reproducibility as well as a lack of biological rationale explaining the observed regulation of certain miRNAs.

Based on the compelling functional data on miR-193a-5p in human and murine HCC [9], we performed an exploratory analysis, aiming at evaluating a diagnostic and/or prognostic role of circulating miR-193a-5p in a cohort of HCC patients undergoing liver resection or LT for early stage disease.

## Materials and methods

### Study design and patient characteristics

We performed this exploratory observational cohort study to evaluate a potential role of circulating miR-193a-5p levels in n = 41 HCC patients undergoing surgical tumor resection (n = 33) or liver transplantation (n = 8). HCC patients who were admitted to the Department of Visceral and Transplantation Surgery at University Hospital RWTH Aachen for tumor resection or LT were prospectively recruited between March 2011 and March 2017. Inclusion criteria were: 1. Age ≥ 18 years; 2. Histologically confirmed HCC; 3. Available blood sample prior to surgery. Exclusion criteria were: 1. Death during or shortly after surgery (<72h) due to surgical complications; 2. Concomitant secondary malignancy. Demographic characteristics of the study population are shown in Table 1. Given the exploratory character of this study, we refrained from sample size and power calculations. The individual decision for or against tumor resection/LT was made in an interdisciplinary tumor board according to internationally recognized standards (e.g. Milan criteria for LT). We collected blood samples prior to surgery, centrifuged them for 10 min at 2000 g, and stored serum samples at −80°C until use. As a control population we included a total of n = 20 healthy, cancer-free blood donors with normal values for blood counts, C-reactive protein, kidney and liver function who are medically examined on a regular basis. The study protocol was approved by the ethics committee of the University Hospital RWTH Aachen, Germany (EK 206/09) and conducted in accordance with the ethical standards laid down in the Declaration of Helsinki. Written informed consent was obtained from the patients.

**Table 1:** Patient characteristics.

	Study cohort
**HCC patients**	41
Gender [%]:	
male-female	70.7–29.3
Age [years, median and range]	66 [42–82]
BMI [kg/m^2^, median and range]	26.36 [17.67–39.18]
Surgical treatment [%]:	
Tumor resection	19.5
Liver transplantation	80.5
HCC etiology:	
Hepatitis B	7.3
Hepatitis C	24.4
NASH	12.2
Alcoholic	12.2
Others	43.9
T-stage [%]:	
T1	25.0
T2	50.0
T3	21.9
T4	3.1
Grading [%]:	
G1	9.7
G2	67.7
G3	22.6
Resection status [%]:	
R0	83.9
R1	16.1
Tumor size [cm, median and range]:	4.65 [1.0–16.8]
ECOG PS [%]:	
ECOG 0	53.7
ECOG 1	41.5
ECOG 2	2.4
ECOG 3	2.4
Deceased during follow-up [%]:	
Yes—No	68.3–31.7

HCC: hepatocellular carcinoma, BMI: body mass index, NASH: non-alcoholic steatohepatitis, ECOG PS: “Eastern Cooperative Oncology Group” performance status.

### miRNA isolation from serum

300 μl serum was spiked with *miScript miRNA mimic SV40* (Qiagen, Germany) for sample normalization. 600 μl *peqGOLD TriFast*^*™*^ (VWR) and 150 μl chloroform were added to the sample and mixed vigorously for 15 sec followed by an incubation at room temperature for 10 min. Samples were centrifuged for 15 min at 12,000 g until complete phase separation. The aqueous phase, containing total RNA, was precipitated with 375 μl 100% isopropanol and 1.5 μl glycogen (Fermentas, St. Leonroth, Germany) overnight at -20°C. After centrifugation at 4°C for 30 min (12,000 g) the pellets were washed once with 70% ethanol and centrifugation at 12000 g, 5 min and 4°C. Precipitated RNA was resuspended in 30 μl RNase free water.

### Semi-quantitative reverse transcriptase PCR (qPCR)

Total RNA was used to synthesize cDNA utilizing miScript Reverse Transcriptase Kit (Qiagen) according to the manufacturer’s protocol, and was resuspended in suitable amounts of H_2_O. cDNA samples (2 μl) were used for semi-quantitative PCR in a total volume of 25 μl using the miScript SYBR Green PCR Kit (Qiagen) and miRNA specific primers (Qiagen) on a PCR machine (Applied Biosystems 7300 Sequence Detection System, Applied Biosystems, Foster City, CA). Data using the 2^-ΔΔCT^ method were presented as relative gene expression. Data were generated and analyzed using the SDS 2.3 and RQ manager 1.2 software packages (Applied Biosystems). For analysis of miR-193a-5 we used the commercially available (Qiagen) primer with the sequence UGGGUCUUUGCGGGCGAGAUGA.

### Statistical analysis

Statistical analyses were performed as recently described [12]. qPCR data is displayed as relative serum levels. Shapiro-Wilk-Test was used to test for normal distribution. Non-parametric data were compared using the Mann-Whitney-U-Test or the Kruskal-Wallis-Test for multiple group comparisons. Correlation analyses were performed using the Spearman’s correlation coefficient. ROC curves were generated by plotting the sensitivity against 1-specificity. Optimal cut-off values for ROC curves were calculated with the Youden-Index method (YI = sensitivity + specificity—1). Kaplan-Meier curves display the impact of realtive miR-193a-5p levels on overall survival (OS). The Log-rank test was used to test for statistical differences between subgroups. The ideal cut-off value for the identification of patients with an impaired OS was calculated using a univariate binary cox proportional hazard model and testing for the minimum p-value in RStudio. The prognostic value of variables was further tested by uni- and multivariate Cox regression analyses. Parameters with a p-value of < 0.250 in univariate testing were included into multivariate testing. All statistical analyses were performed with SPSS 23 (SPSS, Chicago, IL, USA) and RStudio (v1.2.5033, RStudio, Inc., Boston, MA, USA) [13]. A p-value of < 0.05 was considered statistically significant (* p < 0.05; ** p < 0.01; *** p < 0.001).

## Results

### Study population

A total of n = 41 early stage HCC patients either receiving tumor resection (n = 33) or liver transplantation (LT, n = 8) were included into this exploratory analysis. The median age of the study population was 66 years (range: 42–82 years). 70.7% of patients were male and 29.3% female. The underlying disease etiology was distributed as follows: 7.3% hepatitis B, 24.4% hepatitis C, 12.2% NASH, 12.2% alcoholic liver disease and 43.9% others. The median HCC tumor size was 4.65 cm. Table 1 provides a detailed characterization of the study population.

### Circulating levels of miR-193a-5p are upregulated in HCC patients

**Fig 1:**
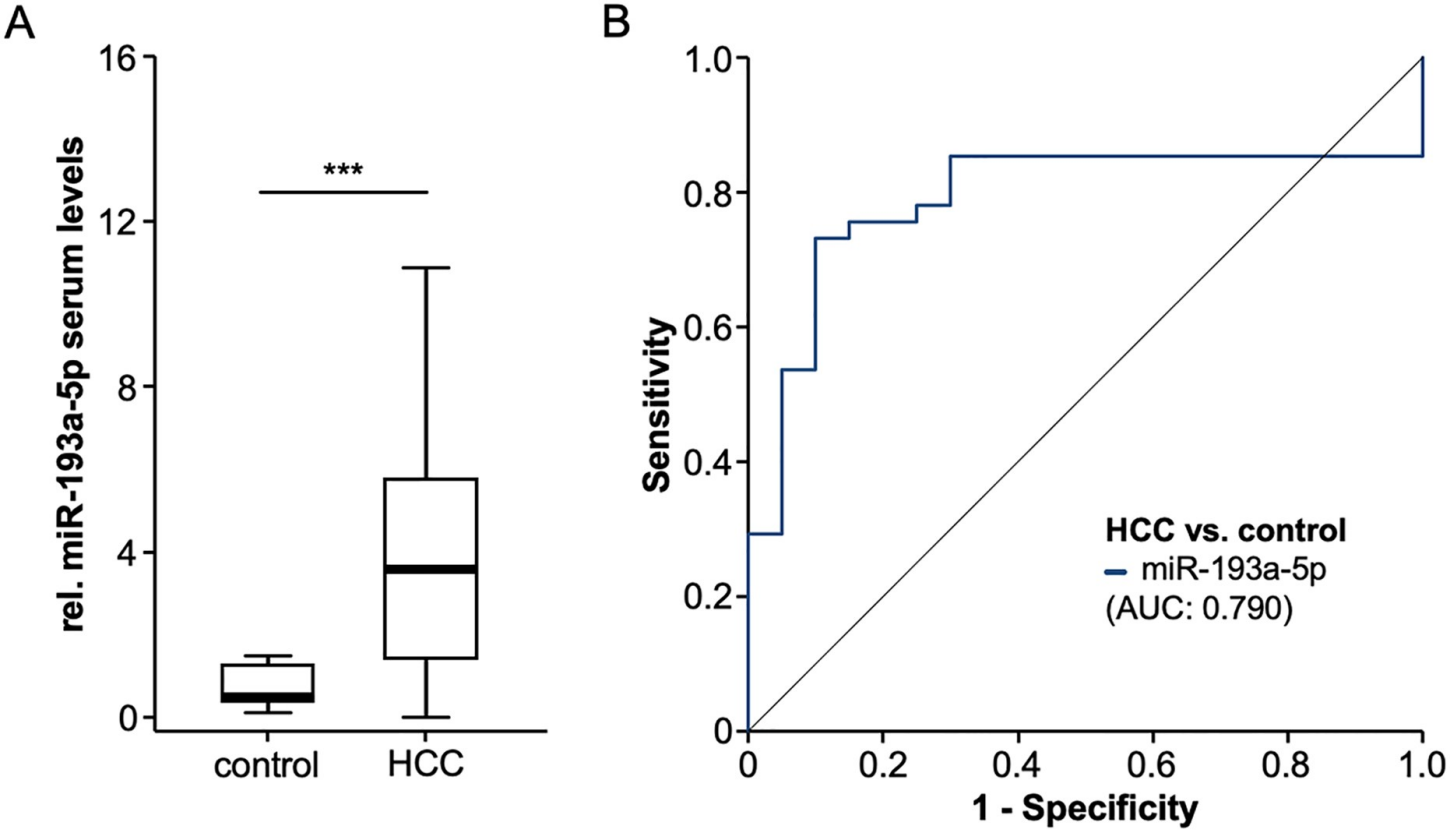
Circulating levels of miR-193a-5p are upregulated in HCC patients. (A) Relative miR-193a-5p levels are significantly upregulated in HCC patients compared to healthy control samples. (B) Relative miR-193a-5p serum levels have an AUC value of 0.790 for the discrimination between HCC patients and healthy controls.

Based on the compelling evidence on a functional of miR-193a-5p in HCC [9], we first analyzed levels of miR-193a-5p in serum samples of HCC patients. Interestingly, this analysis revealed significantly elevated relative miR-193a-5p levels in HCC patients compared to healthy control samples (Fig 1A). We observed a 7.3-fold induction of rel. serum levels of miR-193a-5p in HCC patients with a median level of 0.494 compared to 3.570 in the healthy control samples. In ROC curve analysis, relative miR-193a-5p serum levels showed an AUC value of 0.790 for the discrimination between HCC patients and healthy controls (Fig 1B). At the optimal diagnostic cut-off value of 1.533, relative miR-193a-5p levels had a sensitivity and specificity of 0.732 and 0.9 regarding the diagnosis of HCC.

### Serum miR-193a-5p levels and patients’ characteristics

**Fig 2:**
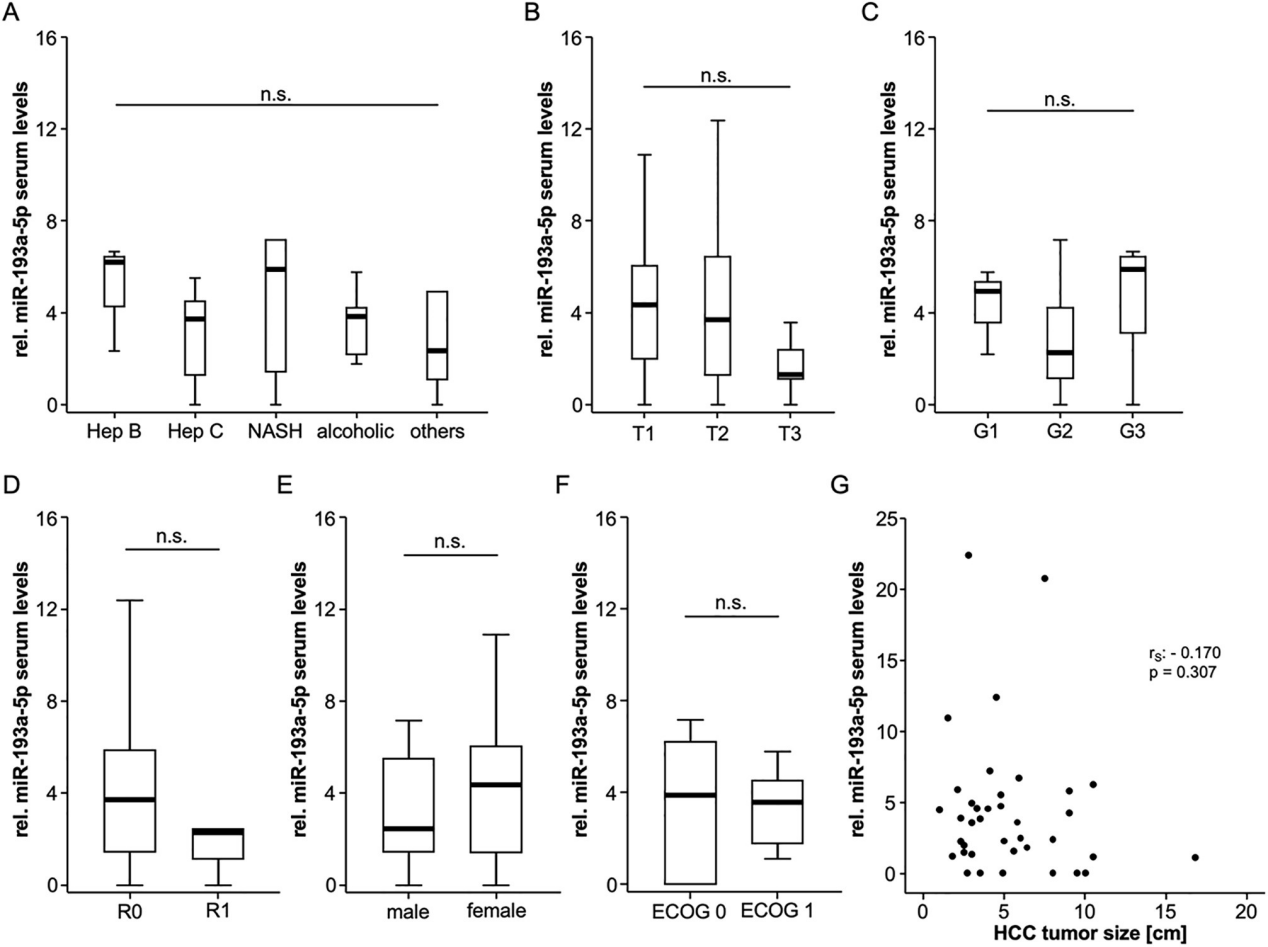
Serum miR-193a-5p levels and patients’ characteristics. Relative miR-193a-5p levels are unaltered between patients with different underlying liver disease etiology (A), tumor stage (B), tumor grading (C), resection status (D), male and female patients (E) or patients with different ECOG performance status (F). Relative miR-193a-5p levels did not correlate with HCC tumor size (G).

We next aimed at identifying potential regulatory mechanisms that drive the upregulation of circulating miR-193a-5p in HCC patients and compared relative circulating levels between several subgroups of patients. Surprisingly, we did not observe significantly altered relative miR-193a-5p levels in patients with different underlying liver disease etiology (Fig 2A), tumor stage (Fig 2B), tumor grading (Fig 2C), resection status (resected patients only, Fig 2D), male and female patients (Fig 2E) or patients with different ECOG performance status (Fig 2F). Moreover, relative miR-193a-5p levels did not correlate with HCC tumor size (Fig 2G).

In a next step, we performed extensive correlation analysis between serum miR-193a-5p and various standard laboratory parameters including markers of liver dysfunction (bilirubin, AST, ALT, GGT, ALP), systemic inflammation (leucocyte count and CRP), renal dysfunction (creatinine), established HCC tumor markers (AFP) as well as hemoglobin and the platelet count (serum levels are displayed in S1 Table). While relative miR-193a-5p levels negatively correlated with the platelet count (r_S_: -0.313, p = 0.047), we were unable to detect a correlation between relative miR-193a-5p levels and the other laboratory parameters (S2 Table).

### Elevated levels of circulating miR-193a-5p predict an impaired outcome

**Fig 3:**
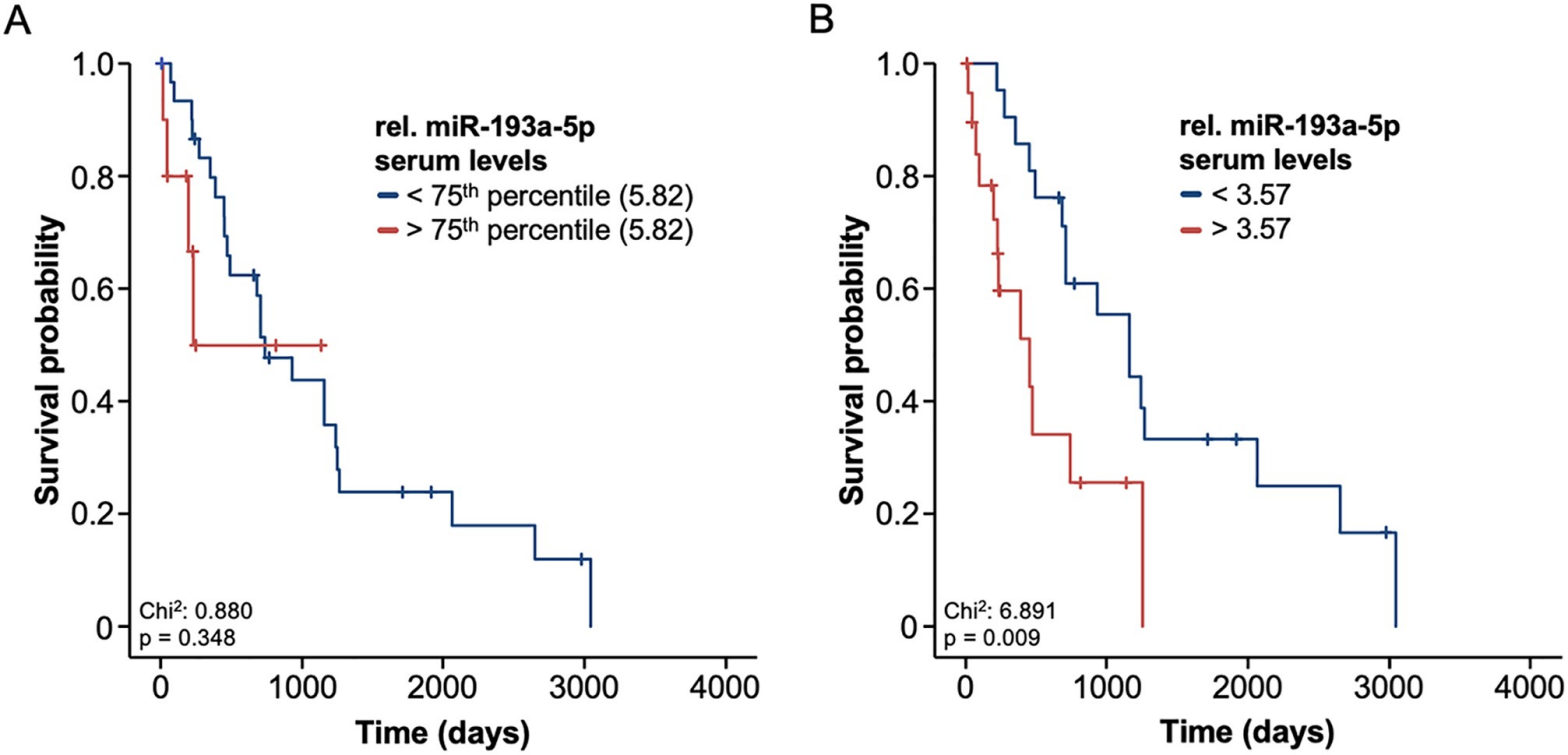
Elevated levels of circulating miR-193a-5p predict an impaired outcome. (A) There is a trend towards an impaired overall survival (OS) in patients with preoperative relative miR-193a-5p levels above the 75^th^ percentile 5.82. (B) HCC patient with a relative miR-193a-5p serum level above the ideal prognostic cut-off value (3.57) have a significantly reduced OS compared to patients with a relative serum level < 3.57. The median OS is 451 days for patients high relative miR-193a-5p levels compared to 1158 days for patients with a relative miR-193a-5p level below the ideal cut-off value.

Based on the prognostic relevance of different circulating miRNAs in the context of HCC [14], we hypothesized that serum levels of miR-193a-5p might also be indicative for the patient’s outcome following surgical therapy. We therefore compared the overall survival (OS) of HCC patients with a very high relative serum levels of miR-193a-5p (above the 75^th^ percentile) and patients with a lower relative miR-193a-5p levels (below the 75^th^ percentile). Here, we observed a trend towards an impaired OS in patients with preoperative relative miR-193a-5p levels above 5.82 (Fig 3A).

Hypothesizing that the 75^th^ percentile might not be the optimal cut-off value, we next established an ideal prognostic cut-off value by using a univariate binary cox proportional hazard model and testing for the minimum p-value. Using this optimal cut-off value (3.57), we observed a significantly reduced OS in patients with a relative miR-193a-5p serum level > 3.57 compared to patients with a relative serum level < 3.57. The median OS was only 451 days for the subgroup of patients showing high relative miR-193a-5p levels compared to 1158 days in those patients with a relative miR-193a-5p level below the ideal cut-off value (Fig 3B). The prognostic relevance of a miR-193a-5p levels above our cut-off value was further confirmed by univariate Cox-regression analysis (HR: 2.936 [1.273–6.776], p = 0.012). To exclude potential confounders with an impact on the OS following tumor resection or LT, we subsequently performed uni- and multivariate Cox-regression analyses including several clinical factors (age, sex, ECOG PS, BMI), pathological parameters (tumor size and tumor grading) as well as several laboratory markers including parameters of systemic inflammation (leucocyte count and CRP), liver (bilirubin and AST) and renal (creatinine) dysfunction, established HCC tumor markers (AFP) and others (Table 2). Importantly, relative circulating miR-193a-5p levels turned out as an independent prognostic marker for OS (HR: 3.708 [1.354–10.159], p = 0.011, Table 2). Together, these data argue for a previously unrecognized prognostic role of circulating miR-193a-5p in HCC patients undergoing surgical tumor resection or LT.

**Table 2:** Uni- and multivariate Cox-regression analysis for the prediction of overall survival.

	univariate Cox-regression		multivariate Cox-regression	
Parameter	p-value	Hazard-Ratio (95% CI)	p-value	Hazard-Ratio (95% CI)
rel. miR-193a-5p levels >3.57 (dichotomized)	0.012	2.936 (1.273–6.776)	0.011	3.708 (1.354–10.159)
Age	0.464	1.015 (0.975–1.057)		
Female sex (dichotomized)	0.504	0.745 (0.315–1.766)		
ECOG PS (0 to 3)	0.331	1.397 (0.713–2.737)		
BMI	0.712	0.986 (0.915–1.062)		
HCC tumor size	0.164	1.071 (0.972–1.180)	0.307	1.061 (0.947–1.190)
Tumor grading (G1 to G3)	0.407	1.518 (0.566–4.067)		
Leukocyte count	0.201	1.110 (0.946–1.304)	0.659	1.044 (0.862–1.265)
Hemoglobin	0.347	0.996 (0.988–1.004)		
Platelets	0.511	1.001 (0.998–1.003)		
Sodium	0.306	1.057 (0.951–1.175)		
Potassium	0.374	1.431 (0.649–3.159)		
AST	0.707	1.002 (0.991–1.013)		
Bilirubin	0.399	0.753 (0.389–1.457)		
AFP	0.589	1.000 (1.000–1.000)		
Creatinine	0.358	1.708 (0.510–5.724)		
CRP	<0.001	1.099 (1.045–1.157)	0.002	1.085 (1.030–1.143)

miR: microRNA, HCC: hepatocellular carcinoma, ECOG PS: “Eastern Cooperative Oncology Group” performance status, BMI: Body-Mass-Index, AST: aspartate transaminase, AFP: alpha-fetoprotein, CRP: C-reactive protein; dichotomized variables are indicated, other variables were continuous.

## Discussion

Hepatocellular carcinoma (HCC) represents the fifth most common cancer worldwide and is associated with a continuously increasing incidence in Europe or North America [15, 16]. In the majority of cases, HCC arises in cirrhotic livers. Despite the recommendation of regular ultrasound surveillance, potentially allowing early diagnosis, only about 30% of all patients present with early disease stage at time of diagnosis [17]. According to Barcelona Clinic of Liver Cancer (BCLC), patients with early disease stages (BCLC 0 or A) are candidates for complete tumor removal e.g. by liver surgery of liver transplantation (LT). However, with the introduction of novel loco-ablative and systemic treatment options it became more obvious that beside these surgical approaches, various other therapeutic options can be offered to patients with early disease stages, especially to those with individual factors arguing against a surgical approach. With all these options available, the individual decision in the interdisciplinary tumour board whether an HCC patient with early disease stage should receive surgical resection / LT or rather be enrolled in a more conservative therapeutic approach is often challenging. At present, the decision for or against surgical treatment is often based on the patient’s liver function, performance status and the technical resectability (including imaging techniques and the assessment of liver function), whereas e.g. aspects of tumour biology are less frequently considered [5]. Therefore, preoperatively available biomarkers could help to better characterize which patients would actually benefit from surgical resection / LT in terms of a personalized therapeutic approach.

Here, we show for the first time that circulating levels of mi-193a-5p are significantly upregulated in patients with early stage HCC. The upregulation was consistent between patient with different underlying disease conditions. Moreover, we could prove that high relative levels of miR-193a-5p are associated with a significantly reduced overall survival (OS) following tumor resection or LT. As such, the median OS was only 451 days for the subgroup of patients showing relative miR-193a-5p levels above the ideal prognostic cut-off value of 3.57 compared to 1158 days in those patients with a relative miR-193a-5p level below the ideal cut-off value. Importantly, multivariate Cox-regression analysis including several clinicopathological parameters as well as markers of organ dysfunction and systemic inflammation revealed circulating miR-193a-5p levels as an independent prognostic factor.

MiRNAs regulate gene expression at post-transcriptional level through a complementary base pairing with the target mRNA, leading to mRNA degradation (in case of perfect complementation) or translation inhibition (in case of imperfect complementation). Based on their tissue-specific expression, their rapid release into the circulation and a remarkable stability in plasma, circulating miRNA are presently scrutinized for their capability as biomarkers for HCC both in a diagnostic and prognostic setting [18, 19]. Measurements of circulating miRNAs might serve as a potential new approach for prompt and non-invasive diagnostic / prognostic screening using real-time PCR. Hence, circulating miR-193a-5p levels were recently discovered as biomarkers in infectious diseases as well as in bladder cancer [20, 21]. We provide evidence that, along with its function as a tumor suppressor in HCC, miR-193a-5p represents a previously unrecognized biomarker in the context of HCC. It should be noted that elevated serum levels of miR-193a-5p in the context of low tissue levels represent only a contradiction of the first view, since similar regulation was described for many miRNAs [22–24] and the specific process interconnecting intra- and extracellular miRNA levels are presently unknown. Recent studies provide evidence that miRNAs are packed into exosomes, arguing for a directed and regulated exchange between the extra- and intracellular miRNA pool [25]. On the other hand, miRNAs might also be passively released during cell death [26]. Thus, the miR-193a-5p represents a biologically plausible biomarker in the context of cancer and in particular HCC. Recently, miR-193a-5p was identified as part of a cell-cycle-targeting network of miRNAs [27]. Administration of nanoparticle-formulated miR-193a-5p inhibited tumor progression in different mouse xenograft models, including three treatment-refractory patient-derived tumors [27]. We and others found a significant down-regulation of miR-193a-5p in tissue samples of different murine mouse models as well as in patients with HCC. Notably, relative serum levels of miR-193a-5p directly correlated with the patients’ survival and indicated an unfavorable tumor biology since patients with high AFP, larger tumor diameter and undifferentiated tumors displayed a further down-regulation of miR-193a-5p compared to others. *In vitro* analysis of our and other groups demonstrated that miR-193a-5p inhibits cell growth, cell migration and formation metastases highlighting the deep integration of miR-193a-5p in the pathophysiology of hepatocarcinogenesis [9, 28]. Despite the currently unknown mechanism of miRNA regulation in the serum, the striking regulation of miR-193a-5p in the serum of HCC patients might have implications for clinical aspects of liver cancer. Therefore, larger patient cohorts with distinct causes of underlying hepatic diseases and differential HCC stages will have to be analyzed to further test the potential of miR-193a-5p levels in the serum as biomarkers for detection and monitoring of HCC.

The present analysis is limited by some aspects. First of all, the exploratory character of our study (n = 41) limits its transferability to the large number of HCC patients treated in clinical routine. Moreover, our analysis only included patients in early disease stages and therefore we can only speculate on whether a similar regulation of miR-193a-5p is also present in patients with more advanced disease stages, receiving e.g. systemic treatments. Moreover, our study did not include alternative treatment approaches such as loco-regional therapies, but only analyzed patients treated with resection / LT for HCC. Thus, we cannot answer the important question whether an HCC patient with an initial serum level above our ideal prognostic cut-off value might have had a similar or even better outcome if treated differently. Given the ongoing studies on adjuvant immunotherapies after resection of HCC [29], a biomarker identifying patients that should receive additional therapy after surgery could be of valuable clinical relevance. Finally, we concentrated on the patients’ OS as a primary endpoint only. Other clinical endpoints such as disease-free survival (DFS) or surgical complications should be included in future confirmatory analyses to fully understand the prognostic relevance of circulating miR-193a-5p in the context of HCC.

In summary, to the best of our knowledge, we show for the first time that circulating miR-193a-5p levels might represent a valuable tool for estimating outcome in patients receiving liver transplantation or tumor resection for early stage HCC. Our data should encourage further multi-center clinical trials including larger patient numbers in different disease stages to provide clearer answers regarding a potential use of miR-193a-5p as a clinical marker in the context of HCC.

## Supporting information

S1 Table Serum levels of laboratory markers. (DOCX)

S2 Table Correlation analysis between relative miR-193a-5p levels and laboratory markers. (DOCX)
