# Supplementary material for: Circulating levels of microRNA193a-5p predict outcome in early stage hepatocellular carcinoma
Source: PLoS One. 2020 Sep 22;15(9):e0239386. doi: 10.1371/journal.pone.0239386 (PMC7508360; doi:10.1371/journal.pone.0239386)
Supplement: S1 Table — (DOCX) [file pone.0239386.s001.docx]

**S1 Table. Serum levels of laboratory markers.**

|  | **HCC patients**  median [range] | **Healthy controls**  median [range] |
| --- | --- | --- |
| rel. miR-193a-5p levels | 3.570 [0-24.140] | 0.494 [0.130-5.442] |
| Leucocyte count [cells/nl] | 6.5 [3.7-13.0] | - |
| Haemoglobin [g/l] | 14.0 [8.5-16.0] | - |
| Platelets [cells/nl] | 165.0 [19.0-754.0] | - |
| Sodium [mmol/l] | 139.0 [126.0-144.0] | - |
| Potassium [mmol/l] | 4.4 [3.3-5.0] | - |
| Bilirubin [mg/dl] | 0.71 [0.26-3.60] | - |
| AST [U/l] | 46.5 [19.0-214.0] | 28.0 [20.0-50.0] |
| ALT [U/l] | 33.0 [7.0-168.0] | 19.0 [5.0-58.0] |
| GGT [U/l] | 133.0 [33.0-794.0] | 17.0 [8.0-52.0] |
| ALP [U/l] | 106.5 [61.0-371.0] | 64.0 [42.0-98.0] |
| Creatinine [mg/dl] | 0.93 [0.46-2.09] | - |
| CRP [mg/l] | 5.15 [0-33.9] | - |
| AFP [µg/l] | 17.6 [2.0-55,368.0] | - |

miR: microRNA, AST: aspartate transaminase, ALT: alanine transaminase, GGT: γ-Glutamyl transpeptidase, ALP: alkaline phosphatase, CRP: C-reactive protein, AFP: alpha-fetoprotein
