# Supplementary material for: Circulating levels of microRNA193a-5p predict outcome in early stage hepatocellular carcinoma
Source: PLoS One. 2020 Sep 22;15(9):e0239386. doi: 10.1371/journal.pone.0239386 (PMC7508360; doi:10.1371/journal.pone.0239386)
Supplement: S2 Table — (DOCX) [file pone.0239386.s002.docx]

**S2 Table. Correlation analysis between relative miR-193a-5p levels and laboratory markers.**

| **Parameter** | **Correlation coefficient (r_S_)** | **p-value** |
| --- | --- | --- |
| AST | -0.135 | 0.406 |
| ALT | -0.184 | 0.369 |
| Bilirubin | 0.078 | 0.628 |
| GGT | 0.253 | 0.125 |
| ALP | -0.019 | 0.914 |
| AFP | 0.301 | 0.135 |
| Sodium | 0.008 | 0.962 |
| Potassium | -0.193 | 0.227 |
| Calcium | -0.175 | 0.279 |
| Hemoglobin | -0.133 | 0.407 |
| Leucocytes | -0.065 | 0.685 |
| Platelets | -0.313 | 0.047 |
| CRP | 0.041 | 0.809 |
| Creatine | -0.018 | 0.911 |

AST: aspartate transaminase, ALT: alanine transaminase, GGT: γ-Glutamyl transpeptidase, ALP: alkaline phosphatase, AFP: alpha-fetoprotein, CRP: C-reactive protein
